# Cardiac transplantation in transthyretin amyloid cardiomyopathy: Outcomes from three decades of tertiary center experience

**DOI:** 10.3389/fcvm.2022.1075806

**Published:** 2023-01-19

**Authors:** Yousuf Razvi, Aldostefano Porcari, Concetta Di Nora, Rishi K. Patel, Adam Ioannou, Muhammad U. Rauf, Ambra Masi, Steven Law, Liza Chacko, Tamer Rezk, Sriram Ravichandran, Janet Gilbertson, Dorota Rowczenio, Iona J. Blakeney, Nandita Kaza, David F. Hutt, Helen Lachmann, Ashutosh Wechalekar, William Moody, Sern Lim, Colin Chue, Carol Whelan, Lucia Venneri, Ana Martinez-Naharro, Marco Merlo, Gianfranco Sinagra, Ugolino Livi, Philip Hawkins, Marianna Fontana, Julian D. Gillmore

**Affiliations:** ^1^Division of Medicine, National Amyloidosis Centre, Royal Free Hospital, University College London, London, United Kingdom; ^2^Cardiovascular Department, Centre for Diagnosis and Treatment of Cardiomyopathies, Azienda Sanitaria Universitaria Giuliano-Isontina (ASUGI), University of Trieste, Trieste, Italy; ^3^Department of Cardiothoracic Science, Azienda Sanitaria Universitaria Integrata di Udine, Udine, Italy; ^4^Imperial College London, London, United Kingdom; ^5^Department of Cardiology, Queen Elizabeth Hospital Birmingham, Birmingham, United Kingdom

**Keywords:** amyloid, transplant, heart failure, TTR–transthyretin, outcome

## Abstract

**Aims:**

Transthyretin cardiac amyloidosis (ATTR-CM) is a progressive and fatal cardiomyopathy. Treatment options in patients with advanced ATTR-CM are limited to cardiac transplantation (CT). Despite case series demonstrating comparable outcomes with CT between patients with ATTR-CM and non-amyloid cardiomyopathies, ATTR-CM is considered to be a contraindication to CT in some centers, partly due to a perceived risk of amyloid recurrence in the allograft. We report long-term outcomes of CT in ATTR-CM at two tertiary centers.

**Materials and methods and Results:**

We retrospectively evaluated ATTR-CM patients across two tertiary centers who underwent transplantation between 1990 and 2020. Pre-transplantation characteristics were determined and outcomes were compared with a cohort of non-transplanted ATTR-CM patients. Fourteen (12 male, 2 female) patients with ATTR-CM underwent CT including 11 with wild-type ATTR-CM and 3 with variant ATTR-CM (ATTRv). Median age at CT was 62 years and median follow up post-CT was 66 months. One, three, and five-year survival was 100, 92, and 90%, respectively and the longest surviving patient was Censored > 19 years post CT. No patients had recurrence of amyloid in the cardiac allograft. Four patients died, including one with ATTRv-CM from complications of leptomeningeal amyloidosis. Survival among the cohort of patients who underwent CT was significantly prolonged compared to UK patients with ATTR-CM generally (*p* < 0.001) including those diagnosed under age 65 years (*p* = 0.008) or with early stage cardiomyopathy (*p* < 0.001).

**Conclusion:**

CT is well-tolerated, restores functional capacity and improves prognosis in ATTR-CM. The risk of amyloid recurrence in the cardiac allograft appears to be low.

## Introduction

Systemic amyloidosis is characterized by extracellular tissue deposition of misfolded fibrillary protein. Amyloid is identified *ex vivo* by apple green birefringence when a tissue biopsy is stained with Congo red dye and viewed under cross-polarized light (1). A variety of normally soluble proteins, known as “fibril precursor proteins,” have been identified which can misfold and self-assemble with an abnormal cross beta-sheet conformation resulting in fibrils that are proteolysis resistant (2). These different “amyloidogenic” proteins form the basis for the classification of amyloidosis. Clinical disease occurs when amyloid deposits disrupt tissue structure and function. Whilst amyloid deposition can occur in almost any organ of the body, cardiac amyloidosis is the established leading cause of mortality in systemic amyloidosis (3).

Transthyretin amyloid cardiomyopathy (ATTR-CM) is the most commonly diagnosed type of cardiac amyloidosis and may be either acquired (ATTRwt-CM) or associated with inheritance of a TTR variant (ATTRv-CM) (2). Historically, treatment for this inexorably progressive and ultimately fatal cardiomyopathy was supportive with meticulous fluid balance and diuretic therapy. However there have been landmark developments in disease-modifying therapy for patients with ATTR amyloidosis. Examples include TTR specific RNA interference or antisense oligonucleotide therapies such as patisiran and inotersen, TTR stabilizers such as tafamidis and acoramidis (4–6), and the CRISPR/Cas9 based gene-editing therapy NTLA-2001 (7). Whilst these agents show promise, to date they appear to slow or potentially halt disease progression rather than being curative. Treatments that bring about an overt clinical improvement and genuinely improve quality of life in patients with advanced ATTR-CM remain elusive.

Cardiac transplantation (CT) for patients with ATTRv-CM was first reported in 2003 (8). However, this was in the context of combined hepatic and CT, the dual objectives being to replace failing cardiac function and remove hepatic production of circulating variant, amyloidogenic TTR which is entirely liver-derived. Hepatic transplantation was mostly undertaken in young patients with hereditary ATTR amyloid polyneuropathy (ATTRv-PN) who typically carried the p.(Val50Met) TTR variant (9). In patients with ATTRv-PN deemed suitable for liver transplantation who had concurrent amyloid cardiomyopathy, a combined hepatic and cardiac transplant would sometimes be offered (10). However, the need for hepatic transplantation in patients with a predominant neuropathic ATTR amyloidosis has largely been superseded by the availability of aforementioned patisiran and inotersen, both of which have been shown to slow or halt disease progression. Consequently, younger patients with advanced ATTR-CM including ATTRwt-CM are increasingly considered for isolated CT (11). There is a paucity of published data on long term outcomes in patients who receive CT for ATTR-CM (11, 12). Allograft amyloid recurrence and accumulation in extra-cardiac organs have remained key concerns in patients with AL amyloidosis receiving CT (either isolated, or combined) (13), and similar concerns regarding potential amyloid recurrence exist in ATTR-CM. Whilst patisiran and inotersen are effective at slowing ATTR amyloid production, they remain licensed only for patients with ATTRv-PN. The risk of cardiac allograft amyloid recurrence is therefore potentially greater in patients, such as those with ATTRwt-CM, who do not routinely receive TTR suppressing agents. We present multicentre outcomes with CT in 14 patients with ATTR-CM evaluated at the UK National Amyloidosis Center and the Department of Cardiothoracic Science, Azienda Sanitaria Universitaria Integrata di Udine, Udine, Italy.

## Materials and methods

### Patients

All patients with ATTR-CM who attended the aforementioned tertiary centers and underwent CT between November 1991 and March 2020 were retrospectively identified from the institutions’ respective databases. Patients who underwent combined liver and heart transplant were excluded. Censor date was 27th July 2022. Patients were considered for referral to a tertiary transplantation center on a case by case basis; typically patients were under aged 65 years with advanced cardiac failure attributable to ATTR-CM and had little or no significant comorbidity.

Patients were managed in accordance with the Declaration of Helsinki, the Declaration of Istanbul and the International Society for Heart and Lung Transplantation Statement on Transplant Ethics. Approval for retrospective analysis and publication of their anonymized data was obtained from the Royal Free London NHS Foundation Trust Ethics Committee (ref 06/Q0501/41).

### Diagnosis

In each case, the diagnosis of ATTR-CM was established according to either validated non-biopsy criteria (14) and/or histological confirmation of cardiac ATTR amyloid deposits with imaging evidence of amyloid cardiomyopathy by echocardiogram and/or CMR. In brief, non-biopsy diagnostic criteria for ATTR-CM consist of echocardiographic and/or CMR imaging evidence suggestive of amyloid cardiomyopathy in together with a Perugini Grade 2 or 3 Tc-DPD scan and absence of a monoclonal gammopathy. Baseline/pre-transplant characteristics were established with biochemical tests which included full blood count, renal function, liver function tests, serum NT-proBNP and high sensitivity troponin T. Serum free light chains (retrospectively from a stored serum sample in patients transplanted before 2000), immunofixation and urine immunofixation were performed in all cases. NAC ATTR Stage was calculated on the basis of NT-proBNP concentration and MDRD eGFR, as previously published (15). Patients were followed routinely at NAC following CT with a full clinical evaluation, biochemical testing and echocardiography; selected cases underwent serial CMR and ^99m^Tc-3,3-Diphosphono-1-2-Propanodicarboxylic Acid (^99m^Tc-DPD) scintigraphy.

### Bone scintigraphy

Patients underwent radionuclide scintigraphy following an intravenous injection of approximately 700 MBq of ^99m^Tc-DPD. Whole body planar and single photon emission computed tomography with a low-dose, non-contrast CT scan (SPECT-CT) images of the heart were acquired 3 h post-injection using low energy, high resolution collimators. Cardiac uptake on all ^99m^Tc-DPD scans was categorized according to the Perugini grading system (16).

### Echocardiography

Image acquisition and analysis was performed by independent, experienced and appropriately accredited echocardiographers in accordance with the latest European Association of Cardiovascular Imaging guidance (17).

### Cardiac magnetic resonance

Cardiac magnetic resonance (CMR) was performed, after 2005, on a 1.5T scanner (Magnetom Aera, Siemens Healthcare, Erlangen, Germany). Localizers and cine imaging with steady state free precession sequences (SSFP) was performed. The contrast agent used was 0.1 mmol./kg of Gadoterate meglumine. Late gadolinium enhancement (LGE) imaging was acquired with magnitude inversion recovery (MAG-IR) and phase-sensitive inversion recovery (PSIR) sequence reconstruction with SSFP read-outs. T1 mapping was performed with the modified look-locker inversion (MOLLI) recovery sequence. T1 mapping was repeated 15 min post-contrast to produce extracellular volume (ECV) maps. All CMRs were analysed by independent consultant cardiologists.

### Histology, proteomics, and immunohistochemistry

All biopsy samples were formalin-fixed and paraffin-embedded. Samples were stained with Congo red by the method of Putchtler, Sweat, and Levine (18). Amyloid fibril type was determined by immunohistochemical staining of amyloid deposits with a range of monospecific antibodies (19). Where necessary, laser microdissection and subsequent proteomic analysis definitively confirmed fibril type (20).

### Genotyping

DNA extracted from blood was amplified by polymerase chain reaction assays and the whole coding region of the *TTR* gene was sequenced to identify any mutations.

## Results

### Pre-transplant characteristics

A total of fourteen cardiac allograft recipients were identified. Patient and disease-related characteristics are shown in Table 1. Twelve of the fourteen recipients were male. Mean age at CT was 59 years, median time from diagnosis to CT was 22 months (IQR 13–29 months). Nine patients had histological evidence of ATTR amyloid deposition on endomyocardial biopsy (EMB) accompanying characteristic cardiac imaging; the remaining patients fulfilled validated non-biopsy diagnostic criteria for ATTR-CM.

**TABLE 1:** Pre-transplant patient and disease characteristics among 14 patients with transthyretin amyloid cardiomyopathy (ATTR-CM) who underwent cardiac transplantation.

Patient	Sex	TTR variant*	Age at diagnosis	Time from diagnosis to CT (months)	NYHA class	NAC ATTR disease stage**	Serum NT-proBNP (ng/L)	LVEF (%)	IVSD (mm)	eGFR (ml/min)	Serum high sensitivity troponin T (ng/L)
1	M	WT	65	11.3	4	2	8778	36	22	64	40
2	M	p.(Val142Ile)	60	1.7	3	2	4609	38	20	56	Unknown
3	M	WT	56	21.1	3	2	4347	35	18	48	37
4	F	p.(Gly73Ala)	43	19.7	3	2	5791	50	20	75	24
5	M	WT	62	48.2	2	1	1057	31	16	90	34
6	M	WT	58	44.4	3	1	3374	56	20	55	81
7	M	WT	57	22.4	2	Unknown	Unknown	Unknown	Unknown	Unknown	Unknown
8	M	WT	59	29.8	3	1	3029	48	14	70	28
9	M	WT	56	7.3	3	2	3434	27	18	90	20
10	F	p.(Ser43Asn)	54	8.7	3	2	5764	39	18	52	80
11	M	WT	61	28.4	3	1	5193	40	18	41	152
12	M	WT	63	20	3	1	2076	39	15	85	102
13	M	WT	63	23	3	2	1345	37	14	43	68
14	M	WT	65	56.4	3	2	5838	27	19	43	43

*WT, wild type TTR genotype **NAC ATTR disease stage is only validated for use at time of diagnosis. Therefore, disease stages at diagnosis, not immediately pre-transplant were used.

Eleven patients had ATTRwt-CM and the remaining 3 had ATTRv-CM associated with the p.(Val142Ile), p.(Gly73Ala), and p.(Ser43Asn) variants respectively. One patient was NYHA functional class IV, eleven patients were NYHA functional class III and the remaining two cases were NYHA functional class II. Median (range) NT-proBNP concentration pre-transplant was 4,202 ng/L (1,057–8,778 ng/L), median (range) left ventricular ejection fraction (LVEF) was 39% (27–56%) and mean (IQR) interventricular septal thickness (IVSd) was 18 mm (14–22 mm). At diagnosis, 8 patients had NAC ATTR stage II disease, 5 had stage I disease and the NAC ATTR stage was not evaluated in the remaining patient. It is notable that patients with NAC ATTR Stage III disease were likely excluded from consideration of CT on the basis of an estimated glomerular filtration rate (eGFR) of < 45 ml/min (19).

### Outcomes

#### Survival

**FIGURE 1:**
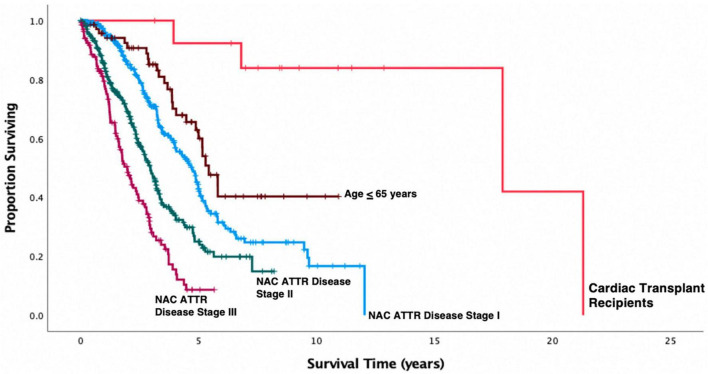
Kaplan-Meier survival curves in 814 patients with transthyretin amyloid cardiomyopathy (ATTR-CM) stratified by NAC disease stage and compared to the cohort who underwent cardiac transplantation. Cardiac transplantation in this small cohort of selected patients was associated with a substantial prolongation of life expectancy measured from date of first diagnosis compared to patients who did not undergo cardiac transplantation (Transplant vs. NAC ATTR stage I, *p* < 0.001; Transplant vs. NAC ATTR stage II, *p* < 0.001; Transplant vs. NAC ATTR stage III, *p* < 0.001) including the cohort of 71 NAC patients who were diagnosed with ATTR-CM under age 65 years (*p* = 0.008).

Post-transplant characteristics are shown in Table 2. Patients were followed up for a median (range) of 66 months (21–233 months). At Censor, 10/14 (71%) patients were alive. Post-CT 1-year survival was 100%, 3-year survival was 92%, and 5-year survival was 90%. Overall estimated post-CT survival in ATTR-CM patients by Kaplan Meier analysis was 17.7 years (95% CI: 13–21 years). Kaplan-Meier survival curves comparing patients who were and were not transplanted, the latter group stratified by NAC ATTR disease stage or by age ≤ 65 years at diagnosis, are shown in Figure 1. Kaplan Meier analysis showed substantially prolonged survival from diagnosis in patients who underwent CT compared to their non-transplanted counterparts regardless of age (*p* = 0.008) or disease severity (*p* < 0.001) at the time of diagnosis.

**TABLE 2:** Outcomes, functional, and disease characteristics in patients with transthyretin amyloid cardiomyopathy (ATTR-CM) who underwent cardiac transplantation.

Patient	LVEF (%)	NYHA functional class	Serum NT-proBNP (ng/L)	eGFR (ml/min)	Significant rejection*	Significant infection**	Renal impairment	Amyloid recurrence***	Outcome (Follow up post-CT, months)	Comments
1	74	II	4627	42	No	No	No	No	Alive (142 m)	Diagnosed with hypertensive heart disease at censor date.
2	53	I	525	86	No	No	Yes–temporary, required dialysis	No	Dead (212 m)	Developed ATTR-PN. Commenced patisiran 210 months post-CT.
3	75	I	182	36	No	No	Yes–post operative CKD	No	Alive (109 m)	
4	60	N/A	382	36	Yes (8 months post CT)	No	Yes–post operative CKD	No	Dead (27 m)	
5	65	I	296	55	Yes (post operative)	No	No	No	Alive (28 m)	
6	56	I	1016	50	No	No	No	No	Alive (57 m)	
7	73	N/A	414	< 15	No	No	Yes–ciclosporin related CKD	No	Dead (233 m)	
8	60	I	611	39	No	No	Yes–post operative CKD	No	Alive (60 m)	
9	59	N/A	905	41	No	No	Yes–required temporary haemodialysis. Subsequent CKD	No	Dead (74 m)	
10	65	I	973	29	No	No	Yes–post operative CKD	No	Alive (29 m)	Developed ATTR-PN. Commenced patisiran 6 months post-CT.
11	50	I	674	46	No	No	Yes–temporary, required dialysis	No	Alive (72 m)	
12	67	I	65	60	No	Yes–CMV****	Yes–temporary	No	Alive (117 m)	
13	63	I	30	37	Yes (post operative)	No	No	No	Alive (60 m)	
14	65	I	620	37	No	Yes–CMV	Yes–post operative CKD	No	Alive (54 m)	

*Significant rejection was defined as rejection requiring treatment as defined by International Society of Heart and Lung Transplantation (ISHLT) criteria.

**Infection was deemed significant if it necessitated hospitalization within 1 month of cardiac transplantation.

***In all cases, amyloid recurrence was assessed for with Tc-DPD scintigraphy and/or endomyocardial biopsy.

****CMV, cytomegalovirus. Data points are the most recently available for all patients. NYHA class is at the time of Censor.

There were no reported episodes of significant post-operative bleeding. Two patients (patient 5 and 13) were successfully treated with intravenous steroids for allograft rejection in the immediate post-operative period. A further patient (Patient 4) required intravenous steroids for grade IIIA allograft rejection 8 months post CT. Two patients were successfully treated in hospital for cytomegalovirus infection during the immediate post-operative period.

At the time of Censor, all but one surviving patient were NYHA functional class I. Patient 1 developed left ventricular hypertrophy 12 years following CT determined to be secondary to hypertensive heart disease. Notably, Tc-DPD scintigraphy in this patient 12 years post CT did not show evidence of cardiac allograft amyloid infiltration. The clinical course of non-surviving patients is available in further detail in Supplementary material 1.

#### Renal impairment

Post-CT renal impairment, including both acute kidney injury thought to be due to perioperative hypoperfusion, and progressive chronic kidney disease (CKD) was common and occurred in 8/14 CT recipients. Three patients required temporary post-operative hemodialysis with two recovering normal renal function, a further four were left with CKD. The final patient developed progressive CKD due to calcineurin inhibitor use 12 years after the CT which progressed to renal failure and hemodialysis dependence 19 years post-CT despite a switch from cyclosporin to sirolimus 5 years prior.

#### Recurrence of amyloid in the cardiac allograft

**FIGURE 2:**
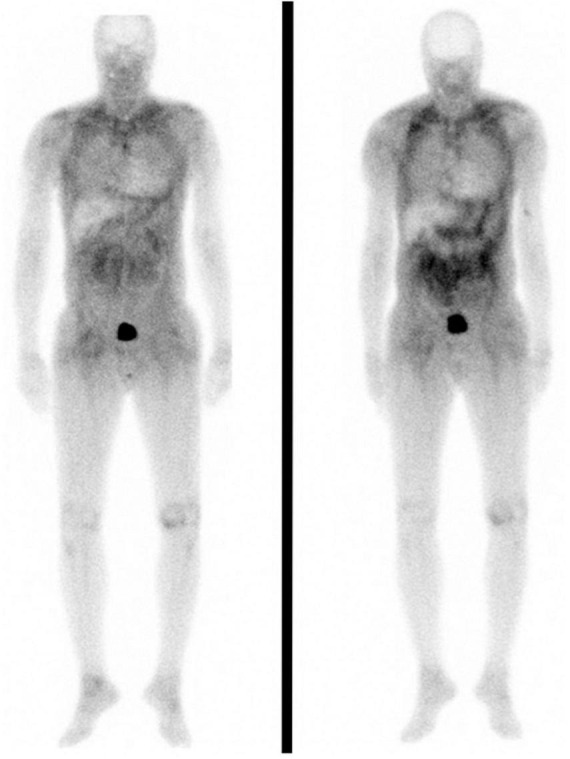
Anterior whole body planar ^99m^Tc-DPD scintigraphy images in patient 2. Left and right panels were obtained seven and ten years post cardiac transplantation, respectively. Both scans are notable for the absence of cardiac uptake despite extensive soft tissue uptake which visibly increased across this 3 year interval.

All patients were assessed regularly at their respective tertiary centers following CT. All patients were assessed for graft amyloid recurrence with Tc-DPD scintigraphy and/or endomyocardial biopsies in combination with CMR imaging. No patient developed recurrent amyloid in the cardiac allograft despite the fact that 12/14 patients did not receive disease-modifying therapy for amyloidosis. Two patients (patient 2 and patient 10), both of whom had ATTRv-CM were commenced on patisiran 210 months and 6 months post-CT respectively following development of mild ATTR-PN. It is notable that patient 2 demonstrated a progressive increase in soft tissue uptake on Tc-DPD scintigraphy post-CT, despite having an allograft EMB that was persistently free from amyloid, no cardiac uptake of Tc-DPD, and no evidence of cardiac amyloidosis on CMR over 17 years after CT (Figure 2).

## Discussion

Here we report multicentre experience in UK and Italy of CT in ATTR-CM. To our knowledge, this is the longest period of follow-up following CT reported to date in ATTR-CM and is the only such study that is multicentre. Both short-term and long-term outcomes were excellent with survival rates comparable to patients undergoing heart transplantation for non-amyloid indications. One-year survival in transplanted ATTR-CM patients was 100%, 3-year survival 92%, and 5-year survival 90%. The International Society for Heart and Lung Transplantation (ISHLT) registry report a 1-year survival rate post-CT of 84% (21). Between 2012 and 2020, the UK National Health Service transplant registry reported CT 1- and 5-year survival rates of 84 and 70% respectively. Whilst our cohort is highly selected and relatively small, Kaplan Meier analysis indicates vastly improved survival compared to unselected ATTR-CM patients, including a UK cohort of 71 un-transplanted patients diagnosed under age 65 years.

Cardiac involvement is the most important determinant of mortality in systemic amyloidosis (3, 22). For example, patients with p.(V50M)-associated ATTR-CM accompanying ATTR-PN carry a prognosis of approximately 5 years, compared to up to ∼15 years in those with p.(V50M)-associated ATTR-PN alone (23). The role of “standard” heart failure therapies including ACE inhibitors, beta blockers and sacubitril/valsartan remains uncertain in ATTR-CM. Some authors argue these aggravate hemodynamic compromise and prohibit effective diuretic therapy (24). The role of SGLT2-inhibitors in ATTR-CM related heart failure is also unknown since ATTR-CM patients were excluded from relevant trials (25). Novel therapeutics such as patisiran, inotersen, and tafamidis, aimed specifically at modifying the inexorably progressive clinical course of ATTR amyloidosis are now in clinical use. However, whilst these agents may be effective at slowing or halting disease progression, there are no data to suggest that they are able to restore normal cardiac function in patients with established ATTR-CM (4–6). Therefore, CT remains the only therapeutic option that has the potential to restore normal cardiac function and quality of life in patients with advanced ATTR-CM.

The first case of CT in amyloid cardiomyopathy was reported in 1984 in a gentleman with cardiac AL amyloidosis (13, 26). Subsequent reports of CT in cardiac amyloidosis highlighted recurrence of amyloid in the cardiac allograft and poor outcomes in comparison to patients undergoing CT for non-amyloid indications, and resulted in amyloid cardiomyopathy being considered an absolute contraindication to CT in some centers (27). However, some case series of CT in amyloid cardiomyopathy consider the condition as a single entity with no differentiation between amyloid types (8, 11, 12), despite the fact that the natural history, organ tropism and prognosis of systemic AL amyloidosis indicate a more aggressive and multi-system disease phenotype than that of ATTR amyloidosis (15, 28). This stark difference in disease natural history coupled with the absence of detectable recurrence of ATTR amyloid in the cardiac allograft of any transplanted patient despite follow up of up to 19 years warrants consideration of the ATTR-CM indication for CT independently from that of cardiac AL amyloidosis. There is strong *in vivo* and *in vitro* evidence that presence of existing amyloid in a tissue promotes ongoing local amyloid deposition (29, 30); this phenomenon, known as “seeding,” was evident in patient 2 who had ongoing accumulation of soft tissue amyloid following CT but did not develop amyloid in his cardiac allograft (confirmed by allograft biopsy 17 years post-CT) which was obviously amyloid naïve at the time of transplantation. One can therefore take encouragement from our cohort that ongoing, unaltered hepatic TTR synthesis does not lead to recurrent amyloid in the cardiac allograft in the short or medium term.

In patients with ATTRv amyloidosis, ATTR-PN accompanying ATTR-CM is a common and important clinical manifestation (2, 4, 23). Prior to the introduction of TTR suppressing and TTR stabilizing agents, liver transplantation to remove the circulating variant transthyretin, was the only available treatment for ATTR-PN. Whilst successful in patients with early-onset p.(V50M)-associated ATTRv amyloidosis who typically have ATTR-PN without ATTR-CM (31), liver transplantation in patients with established ATTRv-CM did not prevent ongoing deposition of wild-type ATTR amyloid deposits on top of the existing template of variant ATTR cardiac amyloid resulting in poor outcomes (32). As a result, combined liver and heart transplantation was performed in some patients with mixed ATTRv-CM and ATTRv-PN (33). However, with the advent of patisiran and inotersen, liver transplantation for ATTRv amyloidosis is now rarely undertaken. A small number of the > 130 known amyloidogenic TTR variants are particularly associated with leptomeningeal ATTR amyloid deposition including p.(Thr69Pro), p.(Leu32Pro), p.(Tyr134Cys), and p.(Gly73Ala) (30, 34–36). Leptomeningeal ATTR amyloid deposits are composed of TTR protein which is synthesized in the choroid plexus rather than liver-derived circulating TTR protein such that patisiran and inotersen are ineffective at preventing neurological disease progression which carries a poor prognosis. Patients being considered for CT with these particular disease-causing variants need careful consideration, highlighted by the poor outcome in patient 4 in our cohort. Whilst patisiran and inotersen specifically target the liver (30, 37), tafamidis has been shown to cross the blood brain barrier (37) and could conceivably stabilize TTR in the cerebrospinal fluid and thereby slow ongoing leptomeningeal ATTR amyloid deposition although this hypothesis remains speculative at the current time (6, 38).

Extracardiac ATTR amyloidosis is well-described in the literature (2). Whilst polyneuropathy, the hallmark phenotypic feature for which TTR gene silencers are licensed in ATTRv (4, 5), is unequivocally caused by amyloid deposits it remains uncertain to what extent amyloid deposits contribute to other common organ manifestations in patients with ATTR amyloidosis such as gastrointestinal disturbance, lumbar canal stenosis and joint pains despite their almost universal presence in the relevant tissue. A recent study reported development of extra-cardiac disease manifestations following CT in patients with ATTR-CM (39) which is entirely consistent with our findings in which two patients were diagnosed with amyloid polyneuropathy 210 and 6 months post CT. However, given the recent availability of TTR gene silencers that effectively halt the progression of ATTR-PN, we would argue that concerns surrounding the possible development of ATTR-PN post CT should not preclude suitable ATTR-CM patients from undergoing CT. With regular monitoring, symptoms of ATTR-PN can be detected at an early stage, and where indicated, TTR gene silencers can be introduced in a timely manner. The orthopedic symptoms which appear to be over-represented in patients with ATTR amyloidosis, such as lumbar canal stenosis and carpal tunnel syndrome, can be effectively alleviated by surgical or non-surgical intervention. Given that cardiac allograft function is typically preserved in ATTR-CM post-CT, such patients are usually able to tolerate general anesthesia from a cardiac perspective.

Our study has limitations. Pre-operative invasive physiological data is not readily available due to the fact transplantation was undertaken in institutions external to the individual amyloidosis centers. Whilst multicentre, the cohort is small and highly selected and we acknowledge the limitations of statistical analyses. Additionally, since disease-modifying therapies have only been available for the past 3–4 years, we are unable to report on outcomes with their long-term use following CT; however, the absence of amyloid recurrence within the cardiac allograft argues against the need for administration of such therapies to protect the cardiac allograft after CT in patients with ATTRwt-CM.

In conclusion, CT in selected patients with ATTR-CM is a robust intervention which restores quality of life and prolongs patient survival. The risk of cardiac allograft amyloid recurrence in the short and medium term appears to be negligible. Longer term follow-up studies will be required to determine whether administration of disease-modifying therapies post-CT provides any additional clinical benefit in patients with isolated ATTR-CM. The authors advise caution when considering the suitability of patients with ATTRv-CM for CT who carry TTR mutations which are known to be associated with important leptomeningeal amyloidosis.

## Data availability statement

The anonymized raw data supporting the conclusions of this article can be made available by the authors upon reasonable request.

## Author contributions

YR and AP conceived the study, carried out data collection, analysis, and manuscript preparation. CD carried out data collection and manuscript review. RP, AI, MR, AM, StL, LC, TR, SR, JaG, DR, IB, NK, DH, HL, AW, WM, SeL, CC, CW, LV, and AM-N contributed to data analysis and manuscript review and editing. MM, GS, UL, and PH contributed with data collection, manuscript preparation, and review. MF and JuG oversaw the study design, data collection, manuscript preparation, review, and final approval. All authors contributed to the article and approved the submitted version.
