# Supplementary material for: Cardiac transplantation in transthyretin amyloid cardiomyopathy: Outcomes from three decades of tertiary center experience
Source: Front Cardiovasc Med. 2023 Jan 19;9:1075806. doi: 10.3389/fcvm.2022.1075806 (PMC9894650; doi:10.3389/fcvm.2022.1075806)
Supplement: Supplementary file 1 [file Table_1.DOCX]

**Clinical course of non-surviving ATTR-CM patients whom underwent cardiac transplantation:**

Patient 4 was a 43 year old female with ATTRv associated with an extremely rare TTR variant, p.(Gly73Ala), known to be associated with development of leptomeningeal amyloid. She presented with predominant ATTR-CM (NAC ATTR Stage II disease, LVEF 50%, IVSd 20mm) and early amyloid polyneuropathy (PND stage 1). There was no evidence of leptomeningeal amyloid on brain MRI. She underwent CT 20 months post-diagnosis with an uneventful post-operative course. Six months post-CT she was admitted to intensive care with significant confusion, drowsiness and ataxia. Initial blood, neuroimaging and cerebrospinal fluid analysis failed to demonstrate a cause for the neurological disturbance and, following a full recovery, she was discharged. She was readmitted ~2 months later with further neurological and cognitive decline. Repeat MRI imaging of the brain demonstrated brain stem enhancement in keeping with leptomeningeal ATTR amyloid. Whilst she partially recovered from this acute on chronic episode, her memory and neurological function continued to decline in a stepwise fashion and neurocognitive assessment two years later showed a verbal IQ of 70, performance IQ of 65 and severely impaired information processing. Given the lack of therapeutic options for leptomeningeal amyloidosis, she was deemed unsuitable for liver transplantation and died one year later (27 months post-CT) from progressive leptomeningeal amyloidosis.

Patient 7 was a 57 year-old male diagnosed with ATTRwt-CM in 1990 conferring NYHA functional class III symptoms. He underwent uncomplicated CT 22 months later and remained well despite evidence of CKD, determined to be secondary to chronic calcineurin inhibitor use, from 2003 which progressed to ESRD for which he started HD in 2009. Due to deteriorating functional capacity, a poor quality of life and poor tolerance of dialysis, he withdrew from renal replacement therapy (RRT) in 2011 and died weeks later.

Patient 9 was a 56 year old male diagnosed with ATTRwt-CM in 2001 (NAC ATTR Stage II disease, LVEF 27%, IVSd 18mm) conferring NYHA functional status class III. CT took place 7 months following diagnosis and was complicated by AKI (from eGFR >90 to requiring 2 weeks of RRT followed by CKD) and 5 years following CT he was admitted with sepsis and acute on CKD which required permanent ongoing RRT. After a 5-month hospital admission during which he became progressively deconditioned, he was discharged to a care home facility. Due to deteriorating quality of life, he withdrew from RRT 7 years post-CT and died shortly afterwards in a hospice.

Patient 2 was a 60 year old gentleman diagnosed with ATTRv-CM in 2004 with NYHA functional class III symptoms and significant LV impairment with an LVEF of 38%. He underwent CT within two months of being diagnosed and made an excellent recovery. Normal functional status was quickly restored. His follow up was generally unremarkable until 17 years post-CT when he began experiencing symptoms of amyloid polyneuropathy. He was assessed by a neurologist and subsequently diagnosed with amyloid polyneuropathy and commenced on patisiran. Two months later he was admitted to hospital with bacterial pneumonia and died aged 78 from septic shock.
